# Considerations Regarding Autologus Blood Injection for Tennis Elbow Tendinopathy

**DOI:** 10.5812/traumamon.19225

**Published:** 2014-08-01

**Authors:** Hayat Ahmad Khan, Younis Kamal, Nazia Hassan

**Affiliations:** 1 Department of Orthopedics, Bone and Joint Hospital, Govt. Medical College, University of Kashmir, Srinagar, India; 2 Govt. Medical College, University of Kashmir, Srinagar, India

**Keywords:** Blood Transfusion, Autologous, Tennis Elbow, Epicondylitis, Lateral Humeral

Dear Editor,

We read with interest the article by Karimi Mobarakeh et al. (1) titled as “Autologous Blood Injection for Treatment of Tennis Elbow”. We would like to congratulate the authors for taking up such a study. However, we have several considerations regarding this manuscript:

1. What was the number of patients who received the local steroid injections before being in the study and also, what was the duration from the last steroid injection before patients were given the autologous blood injection.
2. After injection, the authors state that “Immobilization via a long arm cast was done for 3 weeks”. Unfortunately, the authors have omitted to mention the reasons leading to cast immobilization. Also, there is no information concerning the occurrence of post injection stiffness of elbow when a 3 weeks long arm cast immobilization was maintained.
3. In the Results column, the manuscript states that “The mean duration of symptoms was 7.9 ± 1.3 months. Table 1 shows results of NPS and VAS before and 1, 3 and 6 months after ABI”. However, there is no table available to support the results.
4. Furthermore, in the results column, the authors say that “The level of patient satisfaction on Verhaar scale is shown in Figure 1; 84% of patients showed a high level of satisfaction at the end of the study. None required a second injection, although some of them were obliged to change their activities”. However, no Figure is shown to justify such data.
5. The authors have mentioned that their results were comparable to other studies. However, the studies mentioned in the reference column had used ultrasound guided autologous blood injection for tennis elbow (2). Only one study from the references has used autologous blood injection similar to the present study (3). We would rather suggest the authors to compare their results with comparable studies to justify their implication for the healthcare policy making. We hope our suggestions will be taken with a positive note and the revised manuscript with all missing Figures and Tables will be made available.
